# A Prospective, Randomized, Double-Blind, Split-Face, Comparative Study to Evaluate the Efficacy and Safety of DKL23 and Juvéderm Volift for Correcting Moderate-to-Severe Nasolabial Folds

**DOI:** 10.1093/asj/sjae133

**Published:** 2024-06-14

**Authors:** Mohammad Alimohammadi, Sharon Furman-Assaf, Johan Nilsson

## Abstract

**Background:**

Hyaluronic acid dermal fillers are used for multiple indications, including wrinkle correction and restoration of volume/fullness.

**Objectives:**

The aim of this study was to compare the efficacy and safety of 2 hyaluronic acid products for correcting moderate to severe nasolabial folds (NLFs).

**Methods:**

A prospective, randomized, double-blind, split-face study was undertaken. The subjects’ left and right NLFs were randomly allocated for treatment with DKL23 or Juvéderm Volift. Follow-up was conducted at 1, 3, 6, and 9 months. The changes from baseline on the Wrinkle Severity Rating Scale and the Global Aesthetics Improvement Scale were evaluated. Posttreatment adverse events (AEs) were recorded.

**Results:**

Forty-eight women (median age, 57.0 years) with Type I to VI skin were enrolled. Both treatments showed statistically significant improvement (*P* < .0001) in NLFs according to the Wrinkle Severity Rating Scale score from baseline to each of the time points assessed. The improvement in NLFs was maintained until the end of the study (9 months). Furthermore, the change from baseline to each of the time points assessed was similar between DKL23 and Juvéderm Volift. Investigator- and subject-rated Global Aesthetics Improvement Scale scores showed similar rates of improvement (indicated by the sum of responses of improved, much improved, or very much improved) between the 2 products. The AEs reported in the study were in line with previous and expected experience after injection of hyaluronic acid dermal fillers. The types of AEs, their rates, intensity, and duration were comparable between the 2 products.

**Conclusions:**

DKL23 improved NLF severity from baseline and for up to 9 months, and the results were comparable to the improvement shown by Juvéderm Volift. Treatment was safe and well tolerated.

**Level of Evidence: 2:**

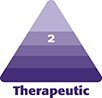

The use of dermal fillers has become one of the most prevalent nonsurgical procedures for aesthetic indications around the world. In the United States, 4.6 million minimally invasive cosmetic procedures utilizing dermal fillers and toxins were estimated to have been performed in 2022.^1^ Hyaluronic acid dermal fillers are used for multiple indications, including wrinkle correction, lip enhancement, restoration of volume/fullness, and scar treatment. Hyaluronic acid fillers have become the most popular treatment for aesthetic correction of nasolabial folds (NLFs). These deep wrinkles or lines, which extend from the bottom of the nose to the corners of the mouth, can become more prominent with age, projecting a fatigued or drawn appearance.^2^

Once injected into the skin, cross-linked hyaluronic acid–based fillers integrate into dermal tissue and bind water molecules which volumize, soften, and hydrate the skin. The gel's physical properties influence how it integrates into tissue after the injection. These properties also affect the product's durability.^3,4^

RadiaNt Derm (DKL23; Dr Korman Laboratories, Kiryat Bialik, Israel) is a sterile, injectable, biodegradable, pyrogen-free, cross-linked sodium hyaluronate 23 mg/mL dermal filler from nonanimal origin in a phosphate-buffered saline solution. The cross-linking agent is 1,4-butanediol diglycidyl ether. The filler does not contain non–cross-linked hyaluronic acid. DKL23 is a CE-marked Class III device intended for the correction of any facial deep skin depressions, face contouring, and volume restorations via facial tissue augmentation.

We conducted a prospective, randomized, double-blind, split-face noninferiority study to compare the efficacy and safety of DKL23 and Juvéderm Volift with lidocaine (Allergan, Inc.) for correcting moderate to severe NLFs and to determine the satisfaction with the product.

## METHODS

### Setting and Participants

The study was conducted at a clinic in Uppsala, Sweden between August 2021 and January 2023. The study was approved by the Ethics Review Authority (approval number 2020-06547 0309) and all subjects signed an informed consent prior to enrolling in the study. The study was registered on clinicaltrials.gov (NCT05021913).

The study population comprised males and nonpregnant, nonbreastfeeding females aged 18 to 65 years with symmetric aging signs in the NLFs with wrinkles classified as Grade 3 (moderate) or 4 (severe) according to the NLF Wrinkle Severity Rating Scale (WSRS).^5^ NLFs were to be of the same grade on the left and right side of the face (ie, approximate bilateral symmetry only). Individuals were excluded from the study if they had porphyria, systemic disorders affecting wound healing, recent significant illness or medical procedures, history of anaphylaxis or severe allergies, known sensitivity to the study products, their components, or lidocaine, immune system disorders, scarring or wounds in the treatment area, facial features that would hinder visualization of treatment, abnormal midface function, or moderate/severe midface asymmetry. Exclusion also applied to those individuals taking certain medications (immunosuppressive therapy, anticoagulants, antiplatelets, thrombolytics, or anti-inflammatory medications) or other substances known to increase coagulation time within 10 days prior to the initiation of study treatment, or those who had recent facial treatments or procedures.

### Choice of Comparator

Juvedérm Volift with lidocaine was chosen as the comparator in this study because it has the same indication as DKL23 and the 2 products have similar physiochemical characteristics; therefore, the products were expected to have a similar clinical impact.

### Randomization, Blinding, Treatment, and Follow-up

A computer-generated randomization list was created with SAS proc plan (SAS v. 9.4). The randomization list contained the subject number and assigned treatment and was kept by the randomizer in a sealed envelope until database lock. The subjects were randomly assigned in a 1:1 ratio to receive DKL 23 mg/mL in the right NLF and Juvéderm Volift with lidocaine in the left NLF or vice versa.

Prior to treatment with the filler, both NLFs were anesthetized with 10 mg/mL lidocaine solution for injection. Then, the treating investigator (unblinded) injected the subjects with up to 2 mL of filler per side (per discretion of the treating investigator) using 27G thin-wall needles provided with the products. Optional touch-up with the same dermal filler in each side was performed after 30 days. Optimal correction was defined as the best possible aesthetic result that could be obtained for an individual study subject, as agreed upon by the treating investigator and the subject. The maximum volume to be injected at touch-up was 1 mL per side. The subjects and the evaluating investigators were blinded to the treatment administered to each NLF.

### Outcome Assessments

Follow-up was performed at 1, 3, 6, and 9 months after the initial injection. At baseline and at each follow-up visit, a total of 3 photographs from 3 angles (left oblique, frontal view, and right oblique) were taken for each subject. Treatment efficacy was evaluated by the change in the NLF WSRS score from baseline to each follow-up as well as by the change from baseline on the Global Aesthetics Improvement Scale (GAIS) score.^6^ These changes were evaluated by the investigator and the subjects, who were blinded to the treatment allocation. The subjects also completed a subject satisfaction questionnaire in which they were asked to indicate their degree of satisfaction with the treatment on a 5-point scale ranging from 1 (very satisfied) to 5 (very dissatisfied). The subjects completed their self-evaluation (subject-rated GAIS and satisfaction questionnaire) independently, prior to the investigator's evaluation of the NLF WSRS and GAIS scores. Responders were defined as subjects who had a ≥1-point improvement on the NLF WSRS for each NLF compared with baseline. Each NLF was assessed separately.

Local tolerability, including symptoms of redness, tenderness, swelling, bruising, and pain, was assessed using subject diaries 14 days after the first treatment session and after touch-up. Any other adverse events (AEs) during treatment and follow-up were recorded throughout the study.

### Statistical Analysis

The data were analyzed with SAS v. 9.4 (SAS Institute, Cary, NC). The primary endpoint was the aesthetic improvement of the NLFs according to the evaluating investigator–rated NLF WSRS score at 6 months. The hypothesis was that DKL23 was noninferior to Juvéderm Volift with lidocaine after 6 months. The margin of noninferiority was set to 0.15 with an assumed ratio between DKL23 and Juvéderm Volift with lidocaine of 1, a significance level of 5%, and a power of 80%.

Categorical variables are reported as count, proportion, and frequency, and continuous variables are reported as mean, standard deviation (SD), median, and range. Changes from baseline within each treatment arm and changes between arms were compared by paired *t*-tests. Findings were considered statistically significant if they were equal to or lower than 5%.

## RESULTS

A total of 48 subjects, all of them female, with a mean [SD] age of 56.4 [5.9] years (range, 44-64 years), were enrolled in the study. Most subjects (98%) were White (Table 1). Five subjects (10.4%) were prematurely withdrawn from the study—4 due to their own decision and 1 was lost to follow-up. The rest (43/48, 89.6%) completed the study, ie, attended the 9-month follow-up assessment.

**Table 1.** Demographic and Baseline Characteristics of the Study Population

Characteristic	Study population (N = 48)
Age (years)	56.4 [5.9] (44-64)
Female	48 (100%)
Fitzpatrick skin type	
Type I	1 (2.1%)
Type II	26 (54.2%)
Type III	19 (39.6%)
Type IV	1 (2.1%)
Type VI	1 (2.1%)
Race	
White	47 (97.9%)
Black or African American	1 (2.1%)
Weight (kg)	74.1 [10.9] (50.1-93.7)

Values are mean [standard deviation] (range) and n (%).

Both products were applied by the linear threading technique. The mean injection volume at the initial treatment was 1.13 [0.28] mL and 1.15 [0.32] mL for DKL23 and Juvéderm Volift with lidocaine, respectively. Thirty-seven subjects (77.1%) received touch-up with a mean injection volume of 0.88 [0.19] mL for DKL23 and 0.88 [0.2] mL for Juvéderm Volift with lidocaine.

### Effectiveness of Treatment

Effectiveness of treatment was evaluated in the per-protocol population (n = 40) which consisted of all randomized subjects who had completed the study who received the initial treatment and had the 6-month assessment without any major protocol deviations that were judged to compromise the analysis of the data. All protocol violations were judged as major or minor prior to database lock. Subjects with a ± 10% change in body weight from baseline at any subsequent visit were excluded from the per-protocol population.

**Figure 1.**
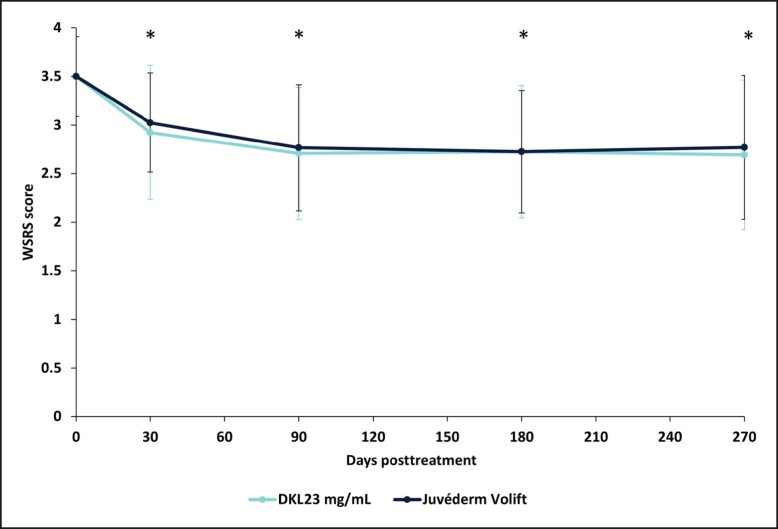
WSRS measured at baseline and each follow-up visit during the study. Each data point depicts the mean and standard deviation of WSRS of 40 subjects treated with DKL23 in one nasolabial fold and Juvéderm Volift in the other nasolabial fold. The WSRS score was evaluated by an investigator who was blinded to the treatment allocation. *P < .0001 for the change in WSRS from baseline. WSRS, Wrinkle Severity Rating Scale.

At baseline, the mean WSRS score was 3.50 [0.41] in both NLFs. The WSRS score statistically significantly decreased from baseline in the NLF treated with DKL23 to 2.93 [0.69], 2.71 [0.68], 2.73 [0.68], and 2.69 [0.77] at 1, 3, 6, and 9 months, respectively, from the initial treatment (*P* < .0001 at all follow-up visits). In the NLF treated with Juvéderm Volift with lidocaine, the WSRS score statistically significantly decreased to 3.03 [0.51], 2.77 [0.65], 2.75 [0.63], and 2.77 [0.74] at 1, 3, 6, and 9 months, respectively (*P* < .0001 at all follow-up visits). The mean absolute change from baseline in the NLF WSRS score for DKL23 at 1, 3, 6, and 9 months was –0.5750, –0.7941, −0.7750, and −0.7949, respectively, as compared with –0.4750, –0.7353, −0.7500, and −0.7179 for Juvéderm Volift with lidocaine. There were no statistically significant differences in the WSRS score between the products at any time point (Figure 1).

**Figure 2.**
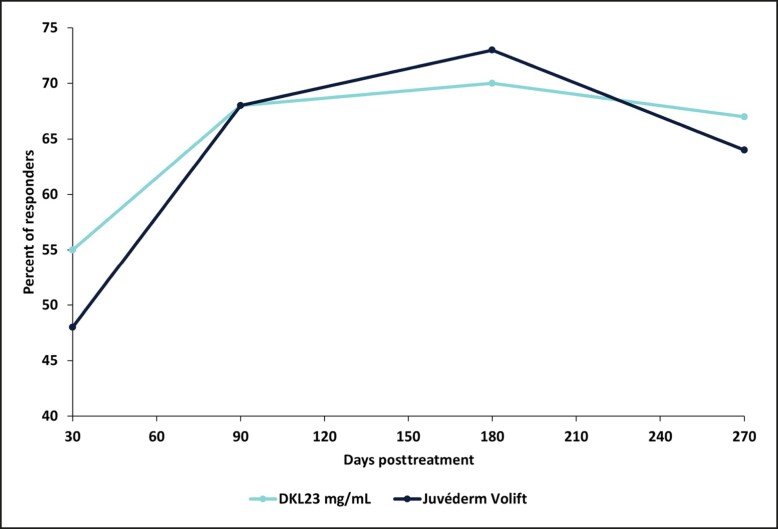
Percentage of responders. Responders were defined as subjects with a ≥1-point change in WSRS score from baseline. Each data point depicts the percentage of responders out of 40 subjects treated with DKL23 in one nasolabial fold and Juvéderm Volift in the other nasolabial fold.

**Figure 3.**
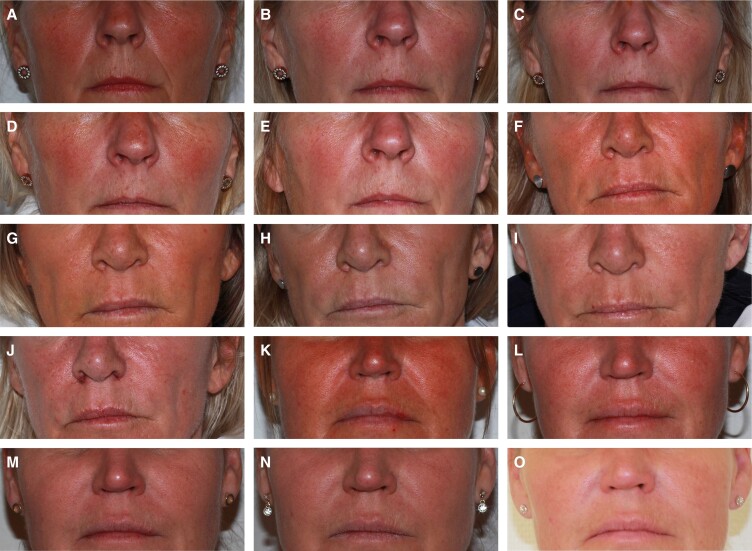
Representative photographs of 3 subjects. (A-E) A 58-year-old woman with Fitzpatrick Type II skin who was treated with DKL23 in the right NLF and with Juvéderm Volift with lidocaine in the left NLF: (A) baseline, (B) 1 month posttreatment, (C) 3 months posttreatment, (D) 6 months posttreatment, (E) 9 months posttreatment. (F-J) A 55-year-old woman with Fitzpatrick Type II skin who was treated with DKL23 in the left NLF and with Juvéderm Volift with lidocaine in the right NLF: (F) baseline, (G) 1 month posttreatment, (H) 3 months posttreatment, (I) 6 months posttreatment, (J) 9 months posttreatment. (K-O) A 51-year-old woman with Fitzpatrick Type III skin who was treated with DKL23 in the right NLF and with Juvéderm Volift with lidocaine in the left NLF: (K) baseline, (L) 1 month posttreatment, (M) 3 months posttreatment, (N) 6 months posttreatment, (O) 9 months posttreatment. NLF, nasolabial fold.

The percentage of subjects with a ≥1-point change in the WSRS score (responders) for the NLF treated with DKL23 compared with the NLF treated with Juvéderm Volift with lidocaine was 55% vs 48% at 1 month after the initial treatment, 68% for both sides at 3 months, 70% vs 73% at 6 months, and 67% vs 64% at 9 months (Figure 2). Representative images of subjects showing the improvement in NLFs from baseline to 9 months posttreatment are shown in Figure 3.

Although the responder rates at 6 months were 70% and 73% for the NLFs treated with DKL23 and Juvéderm Volift, respectively, noninferiority to the comparator could not be statistically proven because the ratio of proportions for responders was 0.9655 (95% CI, 0.7242-1.2871). The statistical testing for noninferiority was based on an implicit assumption of success response rates of 85% for both treatments, but failed to confirm noninferiority due to the lower-than-expected responder rate and the relatively small sample size.

**Figure 4.**
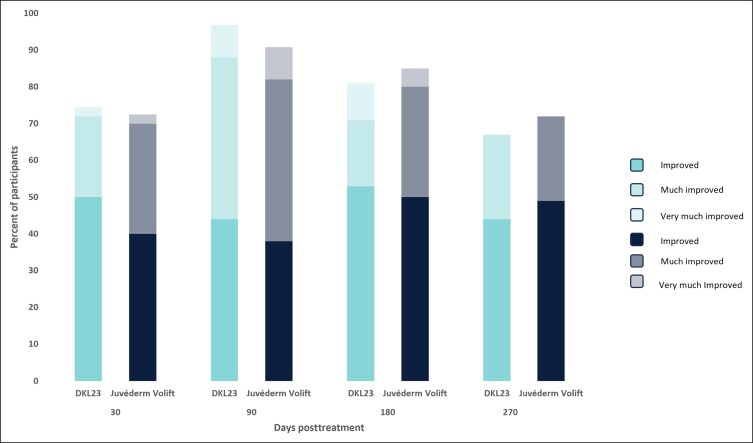
Investigator-rated Global Aesthetics Improvement Scale score during the study. Each bar depicts the percentage of subjects who showed at least improvement from baseline as rated by the investigator.

**Figure 5.**
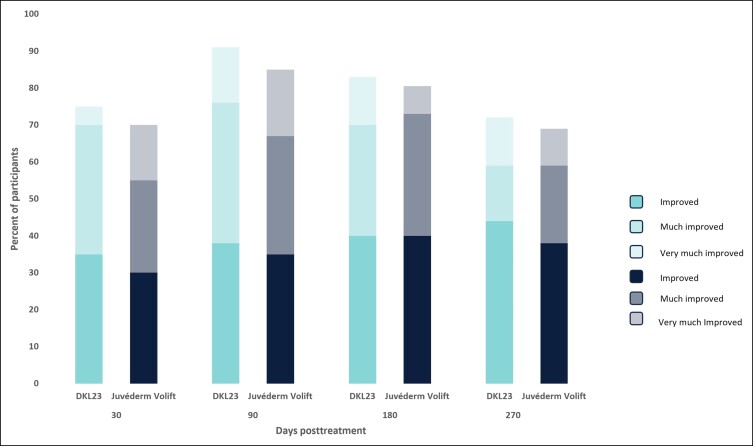
Subject-rated Global Aesthetics Improvement Scale score during the study. Each bar depicts the percentage of subjects who showed at least improvement from baseline as rated by the subject.

At 6 months, 81% of subjects treated with DKL23 and 85% of subjects treated with Juvéderm Volift with lidocaine were rated by the investigator as improved or better vs baseline (Figure 4), and 83% of subjects treated with DKL23 and 80.5% of subjects treated with Juvéderm Volift with lidocaine self-rated themselves as improved or better (Figure 5). At 9 months, 67% of subjects treated with DKL23 and 72% of subjects treated with Juvéderm Volift with lidocaine were rated by the investigator as improved or better vs baseline (Figure 4), and 72% of subjects treated with DKL23 and 69% of subjects treated with Juvéderm Volift with lidocaine self-rated themselves as improved or better (Figure 5).

### Treatment Safety

All 48 subjects experienced at least 1 AE during the study. No serious or unexpected treatment-related AEs were reported during the study.

Most AEs (87.8%) were predefined posttreatment injection site reactions recorded in the subject's diary during the 14 days after initial treatment and touch-up. The most common AEs during the initial treatment were injection-site swelling (96.3% and 81.2% on the side injected with DKL and Juvéderm Volift with lidocaine, respectively), injection-site bruising (81.3% and 75.2% on the side injected with DKL and Juvéderm Volift with lidocaine, respectively), and injection-site tenderness (81% and 67% on the side injected with DKL and Juvéderm Volift with lidocaine, respectively). The rates of predefined AEs were slightly higher on the side injected with DKL compared with the side injected with Juvederm Volift with lidocaine. The AEs were transient and resolved without further treatment, with a median duration of between 1 and 4 days. Among these AEs, bruising had the longest duration for both products. After touch-up, the rates of predefined expected AEs were lower for each product than the rates observed after the initial treatment (Table 2).

**Table 2.** Rate of Expected Posttreatment Adverse Events (Maximum Intensity Reported Per Subject)

	Initial treatment (N = 48)		Touch-up (N = 37)	
	DKL23	Juvéderm Volift	DKL23	Juvéderm Volift
Swelling/edema	46 (96.3%)	39 (81.2%)	34 (91.7%)	33 (89%)
Mild	33 (69%)	28 (58%)	21 (57%)	22 (59%)
Moderate	10 (21%)	9 (19%)	12 (32%)	11 (30%)
Severe	3 (6.3%)	2 (4.2%)	1 (2.7%)	—
Bruising	39 (81.3%)	36 (75.2%)	21 (57%)	20 (54%)
Mild	26 (54%)	22 (46%)	16 (43%)	16 (43%)
Moderate	10 (21%)	12 (25%)	5 (14%)	4 (11%)
Severe	3 (6.3%)	2 (4.2%)	—	—
Tenderness	39 (81%)	32 (67%)	27 (63%)	27 (73%)
Mild	29 (60%)	24 (50%)	20 (54%)	21 (57%)
Moderate	10 (21%)	8 (17%)	7 (19%)	6 (16%)
Redness	37 (77.3%)	32 (66.3%)	22 (60%)	20 (55%)
Mild	33 (69%)	28 (58%)	17 (46%)	15 (41%)
Moderate	4 (8.3%)	4 (8.3%)	5 (14%)	5 (14%)
Pain	24 (50%)	21 (44%)	14 (38%)	13 (36%)
Mild	16 (33%)	15 (31%)	9 (24%)	8 (22%)
Moderate	8 (17%)	6 (13%)	5 (14%)	5 (14%)
Pruritus	10 (21%)	11 (23%)	3 (8%)	3 (8%)
Mild	10 (21%)	11 (23%)	3 (8%)	3 (8%)

Values are n (%).

**Figure 6.**
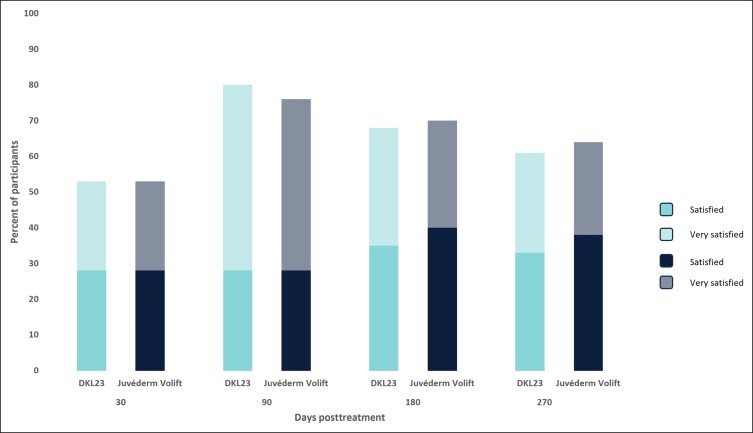
Subject satisfaction. Each bar depicts the percentage of subjects who were satisfied and very satisfied with the products at each follow-up visit.

In addition to the predefined posttreatment injection site reactions, mild implant-site mass was reported by 7 subjects (14.6%). Five of the 7 subjects had the implant-site mass in the NLF area injected with DKL23, and 2 subjects had the implant-site mass in the NLF area injected with Juvéderm Volift with lidocaine. The implant-site mass in 1 subject was removed with hyaluronidase. All other implant-site masses resolved without further treatment. One subject had inflammation of moderate intensity affecting the NLF area injected with Juvéderm Volift with lidocaine, 1 subject had an injection-site nodule of mild intensity affecting the NLF area injected with DKL23, 1 subject had induration of mild intensity affecting the NLF area injected with DKL23, and another subject had mild pruritus, which was not in the treated area, but was assessed as having a possible relationship to the study products. All of these AEs resolved without further treatment. Most subjects were satisfied or very satisfied with both products to a similar extent throughout the study (Figure 6).

## DISCUSSION

Treatment with DKL23 showed statistically significant improvement (*P* < .0001) in NLFs according to the WSRS score from baseline to each of the time points assessed during the study. The improvement in NLFs was maintained until the end of the study (9 months after the initial treatment). Furthermore, the change from baseline to each of the time points assessed was similar for DKL23 and Juvéderm Volift with lidocaine.

Aesthetic improvement in the NLFs was also shown based on GAIS evaluations by the subjects and a blinded evaluating investigator. The highest proportions of NLFs assessed as aesthetically improved were seen at the 3–month follow-up after initial treatment for both DKL23 and Juvéderm Volift with lidocaine. Overall, the proportions of subjects with improvement in mean GAIS score at each assessment time point, and the duration of the improvement, were comparable for the 2 products. According to both the subjects and the evaluating investigator, there were no obvious differences in the degree of improvement (as indicated by the sum of improved, much improved, and very much improved rates) between the 2 products.

Hyaluronic acid fillers improve aesthetic appearance by filling space in skin tissue.^7^ They also hydrate the skin and induce collagen synthesis,^8^ further contributing to the maintenance of cosmetic benefits. Over time, injected hyaluronic acid fillers might be diffused, reabsorbed, degraded, or fragmented in skin tissues.^7^ The split-face design of the study made it possible to compare the products in the same subject, eliminating the effect of subject-related intrinsic factors that may affect the degradation of the products. Indeed, the change in WSRS score over time was similar in both products.

As cross-linked hyaluronic acid dermal fillers have been marketed for almost 30 years, many other studies have also evaluated them for improvement of NLFs. A prospective, open-label, observational, postmarket study that evaluated Juvéderm Volift for the correction of moderate to severe NLFs over 12 months in 65 subjects (mean age, 49 [10] years; range, 26–71 years) showed a response rate of 99% in both NLFs after 1 month and a response rate of 78% in the right NLF and 84% in the left NLF after 9 months. From baseline to Month 9, the mean improvement in mean NLF severity for the right and left NLFs (according to the NLF Severity Scale [NLFSS]) was −1.1 and −1.3, respectively (*P* < .0001 for both NLFs) as assessed by the investigator. The volume of injection to achieve optimum correction, including both initial and touch-up treatment for both NLFs, was a mean of 2.4 [0.5] mL at the initial treatment and 1.6 [0.5] at touch-up after 2 weeks (range including both initial and touch-up treatment, 2.0-6.0 mL; mean, 3.0 [1.0] mL]).^9^ Hence it seems to have been somewhat higher than that in the current study, with a mean of 1.13 [0.28] mL at the initial treatment and 0.88 [0.19] mL at touch-up, and may have affected the observed response rate. The lower volume of the dermal fillers injected in the current study may also be attributed to the lidocaine injection administered to all subjects prior to injection of the dermal fillers.

In a pivotal, prospective, randomized, within-subject–controlled, double-blind study that compared Juvéderm Volift with lidocaine to a control hyaluronic acid filler without lidocaine among 175 Chinese subjects (96.6% female; median age, 42 years; range, 23-65 years), response rates at 6 months using the NLFSS scale were 84.2% and 82.5% for Juvéderm Volift with lidocaine and the control, respectively. Total median injection volumes for combined initial and touch-up treatments for Juvéderm Volift with lidocaine and the control were 1.1 and 1.0 mL, respectively (1.0 and 0.9 mL for initial treatment and 0.4 and 0.4 mL for touch-up treatment, respectively).^10^ In another prospective, multicenter, randomized, within-subject controlled study that compared Juvéderm Volift with control among 126 subjects (95.1% female; 74% White; median age, 54 years; range, 33-83 years) in the United States, the median volume injected at initial treatment was 1.4 mL (range, 0.1-3.0 mL) and the median volume injected at touch-up was 0.7 mL (range, 0.1-1.0 mL). At 6 months, the mean NLFSS scores improved by 1.4 in the NLFs treated with Juvéderm Volift and by 1.3 in the NLFs treated with control (*P* = .097).^11^

It is noteworthy that in the studies mentioned above, the NLFSS score was used to evaluate the change in NLF severity, whereas in the current study, the WSRS score was used. The differences between these scales may have affected the measurements of the absolute change from baseline. Furthermore, the severity of NLFs at baseline may have affected their improvement following treatment. For example, in a prospective, controlled clinical study that compared Belotero Balance Lidocaine and Restylane in 220 Chinese subjects (95.6% female; mean age, 43.4 years) with moderate NLFs according to the WSRS, the response rates at Month 6 based on WSRS assessment (≥1-point improvement compared with screening), as determined by the blinded evaluator, were 62.9% on the Belotero-treated side vs 64.9% on the Restylane-treated side, while at Month 9 the response rates were 49.5% and 50.5%, respectively.^12^

The AEs reported in the study were in line with previous experience after injection of hyaluronic acid dermal fillers.^7-12^ The subject proportions, types of reactions, intensity, and number of days until the AEs had resolved were slightly higher for DKL23, but were overall comparable between the 2 products. This difference can most likely be attributed to the fact that DKL23 is a slightly firmer gel with a higher lifting capacity. Although both NLFs were anesthetized with lidocaine prior to injection of the dermal fillers, the slightly higher rates of posttreatment AEs in the side injected with DKL23 may have been due to the lack of lidocaine in the DKL23 filler. A meta-analysis evaluating the effectiveness and safety of hyaluronic acid fillers with and without lidocaine for NLF treatment, showed that significantly lower pain scores were reported by patients within 30 minutes after injection of fillers with lidocaine compareed to those without lidocaine (mean difference, − 28.83; 95% CI, − 36.38 to − 21.28; *P* < .00001). However, other AEs, including swelling, erythema, bruising, itching, and induration, did not differ between the 2 types of fillers.^13^

This study has several limitations. The first limitation is the study's relatively small sample size of 48 subjects. Although this number was determined as sufficient to show noninferiority to Juvéderm Volift with lidocaine after 6 months, the margin of noninferiority was set to 0.15 with an assumed ratio between DKL23 and Juvéderm Volift with lidocaine of 1, a significance level of 5%, and a power of 80%, and allowing for a 20% dropout rate. Due to the small sample size, and although no statistically significant differences in NLF WSRS were observed between the products, noninferiority could not be shown. Second, the study was designed with a 9-month follow-up, and therefore information on the longevity of the product after this time point is lacking. Additional studies to determine the product's longevity after 9 months are planned. Third, the study aimed to recruit participants 18 to 65 years of age as this is the approved intended population for the filler; however, the participants who fulfilled the inclusion criteria of NLF WSRS scores of 3 or 4 were older, and the final population included mostly women aged 44 to 64 years with Fitzpatrick skin Types II/III. Future studies will include a larger sample size and a more diverse population in terms of age, gender, and skin type.

There are many hyaluronic acid fillers on the market and their selection for treatment depends on the preferences of individual healthcare practitioners. As shown in this study, DKL23 has a good lifting capacity with a similar effect on NLFs to that of Juvéderm Volift with lidocaine—one of the leading hyaluronic acid fillers for this indication. We believe that DKL23 will be preferred by some patients and healthcare practitioners.

## CONCLUSIONS

This prospective, randomized, controlled, split-face study showed that DKL23 improved NLF severity from baseline and for up to 9 months (the end of the study) with comparable improvement to that shown by Juvéderm Volift with lidocaine. Treatment was safe and well tolerated, with similar rates of posttreatment AEs to those of Juvéderm Volift with lidocaine.

## References

[1] Aesthetic plastic surgery national databank statistics 2022. Aesthet Surg J. 2023;43(Supplement_2):1–19. doi: 10.1093/asj/sjad354 38115214

[2] Coleman SR, Grover R. The anatomy of the aging face: volume loss and changes in 3-dimensional topography. Aesthet Surg J. 2006;26(1):S4–S9. doi: 10.1016/j.asj.2005.09.012 19338976

[3] Herrmann JL, Hoffmann RK, Ward CE, Schulman JM, Grekin RC. Biochemistry, physiology, and tissue interactions of contemporary biodegradable injectable dermal fillers. Dermatol Surg. 2018;44(Suppl 1):S19–s31. doi: 10.1097/DSS.0000000000001582 29994947

[4] Fagien S, Bertucci V, von Grote E, Mashburn JH. Rheologic and physicochemical properties used to differentiate injectable hyaluronic acid filler products. Plast Reconstr Surg. 2019;143(4):707e–720e. doi: 10.1097/PRS.0000000000005429 PMC7597953 30921116

[5] Day DJ, Littler CM, Swift RW, Gottlieb S. The wrinkle severity rating scale: a validation study. Am J Clin Dermatol. 2004;5(1):49–52. doi: 10.2165/00128071-200405010-00007 14979743

[6] Narins RS, Brandt F, Leyden J, Lorenc ZP, Rubin M, Smith S. A randomized, double-blind, multicenter comparison of the efficacy and tolerability of Restylane versus Zyplast for the correction of nasolabial folds. Dermatol Surg. 2003;29(6):588–595. doi: 10.1046/j.1524-4725.2003.29150.x 12786700

[7] Qiao J, Jia Q-N, Jin H-Z, et al Long-term follow-up of longevity and diffusion pattern of hyaluronic acid in nasolabial fold correction through high-frequency ultrasound. Plast Reconstr Surg. 2019;144(2):189e–196e. doi: 10.1097/PRS.0000000000005848 PMC6661240 31348336

[8] Savoia A, Landi S, Baldi A. A new minimally invasive mesotherapy technique for facial rejuvenation. Dermatol Ther (Heidelb). 2013;3(1):83–93. doi: 10.1007/s13555-012-0018-2 23888258 PMC3680640

[9] Sattler G, Philipp-Dormston WG, Van Den Elzen H, et al A prospective, open-label, observational, postmarket study evaluating VYC-17.5L for the correction of moderate to severe nasolabial folds over 12 months. Dermatol Surg. 2017;43(2):238–245. doi: 10.1097/DSS.0000000000000939 28165349 PMC5414733

[10] Xie Y, Li Q, Gao Z, et al Juvéderm volift (VYC-17.5L), a hyaluronic acid filler with lidocaine, is safe and effective for correcting nasolabial folds in Chinese subjects. Clin Cosmet Investig Dermatol. 2022;15:237–245. doi: 10.2147/CCID.S344350 PMC8859541 35210801

[11] Monheit G, Beer K, Hardas B, et al Safety and effectiveness of the hyaluronic acid dermal filler VYC-17.5L for nasolabial folds: results of a randomized, controlled study. Dermatol Surg. 2018;44(5):670–678. doi: 10.1097/DSS.0000000000001529 29701621 PMC6221389

[12] Li W, Li B, Hofmann M, Klein G, Xie H. A multicenter noninferiority study comparing safety and effectiveness of hyaluronic acid fillers for correction of nasolabial folds in Chinese subjects. Plast Reconstr Surg Glob Open. 2023;11(2):e4810. doi: 10.1097/GOX.0000000000004810 36845861 PMC9945413

[13] Wang C, Luan S, Panayi AC, Xin M, Mi B, Luan J. Effectiveness and safety of hyaluronic acid gel with lidocaine for the treatment of nasolabial folds: a systematic review and meta-analysis. Aesthetic Plast Surg. 2018;42(4):1104–1110. doi:10.1007/s00266-018-1149-3 29740661

